# Solving the Combined Heat and Power Economic Dispatch Problem in Different Scale Systems Using the Imperialist Competitive Harris Hawks Optimization Algorithm

**DOI:** 10.3390/biomimetics8080587

**Published:** 2023-12-04

**Authors:** Amir Nazari, Hamdi Abdi

**Affiliations:** Electrical Engineering Department, Engineering Faculty, Razi University, Kermanshah 67144-14971, Iran; amirnazaric@gmail.com

**Keywords:** combined heat and power units, large-scale power system, optimization, imperialist competitive Harris hawks optimization, meta-heuristic algorithm

## Abstract

The aim of electrical load dispatch (ELD) is to achieve the optimal planning of different power plants to supply the required power at the minimum operation cost. Using the combined heat and power (CHP) units in modern power systems, increases energy efficiency and, produce less environmental pollution than conventional units, by producing electricity and heat, simultaneously. Consequently, the ELD problem in the presence of CHP units becomes a very non-linear and non-convex complex problem called the combined heat and power economic dispatch (CHPED), which supplies both electric and thermal loads at the minimum operational cost. In this work, at first, a brief review of optimization algorithms, in different categories of classical, or conventional, stochastic search-based, and hybrid optimization techniques for solving the CHPED problem is presented. Then the CHPED problem in large-scale power systems is investigated by applying the imperialist competitive Harris hawks optimization (ICHHO), as the combination of imperialist competitive algorithm (ICA), and Harris hawks optimizer (HHO), for the first time, to overcome the shortcomings of using the ICA and HHO in the exploitation, and exploration phases, respectively, to solve this complex optimization problem. The effectiveness of the combined algorithm on four standard case studies, including 24 units as a medium-scale, 48, 84, units as the large-scale, and 96-unit as a very large-scale heat and power system, is detailed. The obtained results are compared to those of different algorithms to demonstrate the performance of the ICHHO algorithm in terms of better solution quality and lower fuel cost. The simulation studies verify that the proposed algorithm decreases the minimum operation costs by at least 0.1870%, 0.342%, 0.05224%, and 0.07875% compared to the best results in the literature.

## 1. Introduction

Today, energy saving and reducing pollutants are the most important concerns, mainly in industrialized societies. Therefore, technologies that play an effective role in this regard are given serious attention. The amount of fossil energy is finite and its use causes significant environmental pollution; therefore, the optimal management of these resources is very important in electrical energy production. One of the attractive methods for optimizing energy consumption is the use of the combined heat and power (CHP) unit, which simultaneously produces electricity and heat in a single system [1]. In the CHP unit, due to the simultaneous production of two types of energy, the energy efficiency increases up to 90% [2], which significantly increases the efficiency of about 30% of traditional thermal power plants [3]. Also, in CHP units, the operating costs are reduced by 10–40% [4], and the amount of pollution production is decreased up to 13–18% [5].

Reducing operational costs due to the high costs of building new power plants and transmission lines is always one of the main and important issues in the operation, and planning of power systems. The planners, and operators of this largest human-made system are always looking to reduce various operating costs by using efficient optimization techniques. Therefore, power system optimization, especially in the power generation sector, considering new facilities, such as CHP technology, is an important and interesting topic. By using more powerful optimization methods, and finding lower operating costs, the cost of power generation will be significantly reduced, and cost savings can be used to develop new projects and replace old equipment with new, and modern equipment.

In an integrated framework, where the use of thermal power plants is inevitable, using the CHP units is a suitable solution to increase power generation efficiency. This gives rise to the combined heat and power economic dispatch (CHPED) problem [6]. In this new framework, thermal power plants, thermal boilers, and CHP units are considered to supply electric and thermal loads [7]. The CHPED problem aims to minimize the total cost of operating the ONN units, satisfying all equality and inequality constraints [8]. The main challenges that complicate the CHPED concept, include the valve point loading effect (VPLE) of thermal power units [9] and the power and heat independency of CHP units, known as the feasible operating region (FOR) [10].

### 1.1. A Brief Review of the Proposed Methods

In [8], a comprehensive review of some heuristic optimization algorithms applied to CHPED problem is presented, by providing the comparative results for 4-unit, 5-unit, 7-unit, 24-unit, and 48-unit test systems. Although the presented review contains useful material and directions for future research, no classifications are given, and the scope of the study was limited to use only some heuristic optimization algorithms.

Many studies have been done on the CHPED issue by implementing different approaches and techniques. They are mainly classified into three main categories, namely classical, or conventional methods, stochastic search-based techniques (evolutionary or heuristic algorithms), and hybrid approaches. As an important note, and despite a vast diversity of optimization algorithms proposed and used to solve the CHPED problem, finding better results for this problem in terms of accuracy and run-time, remains a very challenging issue and is the subject of ongoing research.

#### 1.1.1. Classical, or Conventional Methods

The first category includes the mathematical methods like Lagrangian relaxation (LR) [11], two-layer LR [12], benders decomposition (BD) [13], nonlinear mixed-integer programming (NLMIP) [14], branch and bound (B&B) algorithm [15], semidefinite programming (SDP) method [16], dual partial-separable programming method [17]. Such methods are fast and robust, and give almost similar cost values, but the corresponding burdens and runtimes are different [18]. These methods are derivative-based techniques and are highly sensitive to the starting point and nature of the objective function. So the results obtained may not be global or even close to the global optimal solution [19].

#### 1.1.2. Stochastic Search-Based Techniques

The second category is stochastic search-based techniques (evolutionary or heuristic algorithms), which are widely used in optimization problems, including the CHPED problem. These methods can address the complexities of the CHPED problem, such as value point loading effects (VPLE) [20].

The presented methods/algorithms in this category can be classified in evolutionary algorithms (EAs), swarm intelligence-based algorithms, human-based algorithms, and physics-based algorithms, as follows:

- EAs: differential evolutionary (DE) [21], evolutionary programming (EP) [22], neighborhood-based differential evolution algorithm with direction induced strategy (NDIDE) [23], genetic algorithm (GA) [24], real-coded genetic algorithm with random walk-based mutation (RCGA-CRWM) [25], crisscross optimization algorithm (COA) [26], and stochastic fractal search (SFS) algorithm [27]
- Swarm intelligence-based algorithms: grasshopper optimization algorithm (GOA) [6], bee colony optimization (BCO) algorithm [28], adaptive cuckoo search with differential evolution mutation (ACS-DEM) [29], wall optimization algorithm (WOA) [30], cuckoo search algorithm (CSA) [31], group search optimization (GSO) [32], wild goats algorithm (WGA) [33], particle swarm optimization (PSO) [34], firefly algorithm (FA) [35], invasive weed optimization (IWO) algorithm [36], marine predators algorithm (MPOA) [37], and artificial bee colony (ABC) [38]
- Human-based algorithms: exchange market algorithm (EMA) [2], social cognitive optimization algorithm with tent map (TSCO) [39], imperialist competitive algorithm (ICA) [40], heap-based optimizer algorithm (HBOA) [41], supply–demand optimization (SDO) algorithm [42], and Kho-Kho optimization (KKO) [43]
- Physics-based algorithms: harmony search algorithm (HS) [44], gravitational search algorithm (GSA) [45], heat transfer search algorithm (HTSA) [46], and the Rao-I algorithm [47].

Also, some improved or enriched versions of EAs applied to the CHPED problem fall into this category. These new algorithms are proposed to prevent the convergence of the original EAs to local optima and increase the convergence speed [20]. Biogeography-based learning particle swarm optimization (BLPSO) [5], PSO algorithm with time-varying coefficients [48], improved PSO (IPSO) [49], selective particle swarm optimization (SPSO) [50], time-varying acceleration coefficients PSO (TVAC-PSO) [51], improved group search optimization (IGSO) [20], improved marine predators algorithm (IMPOA) [37], improved Mühlenbein mutation (IMM) [52], improved GA (IGA) [53], self-adaptive real-coded genetic algorithm (SARGA) [54], improved artificial bee colony (IABC) algorithm [55], society-based grey wolf optimizer (SGWO) [56], cuckoo optimization algorithm with penalty function (PFCOA) [57], and effective cuckoo search algorithm (ECSA) [58] are some examples of this type of optimization techniques which were applied to the CHPED problem. As these techniques are derivative-free, they do not need a good starting point, and can escape from local minima solutions [53]. These algorithms cannot guarantee finding the optimal solution, do not provide meaningful measurement regarding the distance from the global optima, and suffer from premature convergence [18].

#### 1.1.3. Hybrid Optimization Methods

Optimally solving the CHPED problem using purely EAs or classical techniques is very difficult or even impossible, especially by considering the different objective functions, and various constraints. As an effective and appropriate solution, two significant categories of hybrid methods, including hybrid classical and EAs, and hybrid EAs (two or more EAs) have been addressed in the literature.

- Hybrid classical and EAs: the combinatorial of differential evolution (DE) with sequential quadratic programming (SQP) [59], Lagrange relaxation-based alternating iterative (AI) algorithm [60], and augmented Lagrange–Hopfield network method [61].
- Hybrid EAs: the combination of harmony search (HS) algorithm and PSO (IHSPSO) [62], integrated civilized swarm optimization (CSO) and Powell’s pattern search (PPS) [63], hybrid HS and Nelder-Mead (NM), called the NM-HS algorithm [64], integrated genetic algorithms and tabu search [65], hybrid heap-based and jellyfish search algorithm (HBJSA) [66], real coded genetic algorithm with improved Mühlenbein mutation (RCGA-IMM) [52], hybrid modified grasshopper optimization algorithm (MGOA) and the improved Harris hawks optimizer (IHHO), known as MGOA-IHHO [67], hybrid chameleon swarm algorithm (CSA) and mayfly optimization (MO), named CSMO [68], fuzzy adaptive ranking-based crow search algorithm (FRCSA) with modified artificial bee colony (ABC), known as (FRCSA-ABC) [69], weighted vertices-based optimizer (WVO) and PSO algorithm, or WVO–PSO [69], hybrid time varying acceleration coefficients-gravitational search algorithm-PSO (hybrid TVAC-GSA-PSO) [70], hybrid firefly and self-regulating PSO (FSRPSO) [71], bat algorithm (BA) and artificial bee colony (ABC) with chaotic based self-adaptive (CSA) search strategy (CSA-BA-ABC) [72], self-adaptive learning with time varying acceleration coefficient-gravitational search algorithm (SAL-TVAC-GSA) [73], fast non-dominated TVAC-PSO combined with EMA [74], and adaptive inertia weight PSO (AIWPSO) [75].

It should be noted that there are other heuristic methods, known as hyperheuristics, were suggested to deal with the complex optimization problems. They comprise a set of methods that are motivated (at least in part) to automate the design of heuristic methods to solve the hard computational search problems, and refer to a search technique or learning mechanism to select or generate heuristics to solve computational search problems [76]. The main hyper-heuristic categories are heuristic selection and heuristic generation. Some early approaches developed before 2000 are automated heuristic sequencing, automated planning systems, automated parameter control in evolutionary algorithms, and automated learning of heuristic methods [77]. Further details on this subject can be found in [76,77,78].

### 1.2. The Constraints of the CHPED Problem, and Case Study Systems

In this sub-section, the constraints of the CHPED problem, and different case study systems are addressed.

#### 1.2.1. Problem Constraints

As mentioned, the CHPED is a complex and very nonlinear problem that includes different equality and inequality constraints. The main inequality constraints which are mainly related to thermal power plants consist of VPLE, prohibited operating zones (POZs), and ramp-rate limits (RRLs). Also, the FOR is considered a very important inequality constraint of the CHPED problem. On the other hand, modeling the transmission losses (TLs) is a more challenging issue. Table 1, presents a brief summary of this subject for some limited cases.

#### 1.2.2. Case Study Systems

The simulated case study systems are mainly categorized into four different sizes small, medium, large, and very large scales. The small-scale systems are including 4, 5, and 7 units. The 11-unit, and 24-unit test systems are considered as medium-scale systems. The 48-unit, and 84-unit systems are grouped as large-scale test systems, and finally 96, and more units’ test systems are considered very large-scale systems. Table 1 presents some detailed data on this issue.

### 1.3. Paper Contributions

The two main challenges of this problem are modeling the VPLE of POUs, and dependence of heat and power generation in CHP units. These cases create multiple local minima, and turn the problem into a highly nonlinear, non-convex, and non-smooth constrained optimization problem. The literature confirms that the vast majority of optimization methods can inherently handle only unconstrained problems [82]. Also, gradient-, or derivative-based techniques, usually become easily trapped in local minima. On the other hand, the use of heuristic methods has disadvantages, such as, difficulty in initializing the initial population, high run-time, less guaranteed convergence, a large number of setting parameters, high sensitivity to setting parameters, and the need for many iterations for convergence [19]. Considering the operation of these units for most of the year, any slight reduction in the final solutions of the problem will lead to cost savings in the range of thousands and even millions of dollars per year. All these cases require us to use more powerful algorithms to optimize the problem.

The ICA has already been used to solve the CHPED problem, in small-, medium-, and large-scale heat, and power systems, where the obtained results were superior to other algorithms. However, the literature confirms the poor performance of this algorithm in the exploitation phase. To overcome this weakness, the combination of this algorithm with the HHO algorithm has been used.

In this work, for the first time, the ICHHO algorithm, as the combinatorial version of ICA, and HHO is applied to solve the aforementioned problem in medium-, large-, and very large-scale combined heat and power systems. It should be noted that in our previous research [81], the ICHHO was introduced and applied mainly to multi-zone power and heat systems, on some small- and large-scale systems, and the performance of the algorithm was tested on the standard cases of five, seven, and forty-eight units in the multi-zone combined heat and power systems. The main objective of that reference was to determine the optimal operation points of the CHPED problem in the small multi-zone combined heat, and power systems, which was initially used to test the efficiency of the proposed ICHHO algorithm.

However, the main contribution of this new research is to solve the CHPED problem in single-zone combined heat and power structures, in different scales of medium, large, and very large, to verify the effectiveness and performance of the proposed algorithm. The studied systems in this new work are larger than the previously analyzed systems in terms of variables, which can completely challenge the ability of the proposed algorithm in solving large-scale, nonlinear, and complex problems. The most important innovation of this paper is reducing the generation costs in different complex heat and power systems, which has significantly reduced the annual operation costs compared to other research. For this purpose, this research investigates the CHPED problem on the 24, 48, 84, and 96-units in single-zone systems, by using some comparative analysis. To attain this goal four different case studies are considered, modeling the VPLE of power-only units (POUs) in all cases. Initially, to investigate the ICHHO algorithm performance, a 24-unit as medium-scale system which consists of 13 POUs, 6 cogeneration units, and 5 heat-only units (HOUs) is investigated. The results from these systems show the satisfying accomplishment of the suggested algorithm to handle the CHPED issue. In addition, two standard large-scale test systems, including 48, and 84 units, are also studied. The 48-unit system includes 12 CHP units, 26 POUs, and 10 HOUs. The second large-scale system, 84-unit, includes 40 POUs, 24 CHP units, and 20 HOUs. Also, the very large-scale system, 96-unit is including 52 POUs, 24 CHP units, and 20 HOUs. The results of the ICHHO algorithm show that this algorithm is predominant from the aspect of operation cost to other algorithms.

The main novelties of this research are as follows:

- Providing a short review of the proposed methods to solve the CHPED problem.
- Proposing the ICHHO algorithm, to overcome the shortcomings of ICA, and HHO in the exploitation, and exploration phases, respectively to increase the performance of the hybrid algorithm.
- Utilizing the ICHHO, for the first time to medium-, large- and very large-scale combined power and heat systems, by modeling the VPLE, and FOR of CHPs.
- Investigating the algorithm performance on the studied cases, and comparing the obtained results with other techniques in the literature.
- Confirming the algorithm’s ability to find the optimum points of the CHPED problem in large-scale systems.

### 1.4. Paper Structure

The rest sections of this research are structured as follows. In Section 2, the mathematical modeling of the problem is explained. Section 3 addresses the ICHHO algorithm, and the applied formwork to the CHPED problem. The results of the ICHHO application on different case studies and their comparison with other algorithms are addressed in Section 4. Ultimately, the main conclusions are detailed in Section 5.

## 2. Mathematical Formulation of CHPED Problem

The CHPED problem involves determining the optimal generations of POUs, HOUs, and CHP units, satisfying all of the practical constraints to minimize the operation costs. The problem should be considered by modeling the VPLE, generation capacity limits of different units, and the interdependence of heat and power of CHP units. In this section, the mathematical modeling of the CHPED problem is presented.

### 2.1. Objective Function

The CHPED problem is an optimization problem, aiming to minimize the fuel cost of committed units in terms of ($/h) and is expressed as Equation (1):

(1) $$\min\;\mathrm{TC}=\overset{N_{p}}{\underset{i=1}{\sum }}C_{pi}\left(P_{i}\right)+\overset{N_{c}}{\underset{i=1}{\sum }}C_{ci}\left(O_{i},H_{i}\right)+\overset{N_{h}}{\underset{i=1}{\sum }}C_{hi}\left(T_{i}\right)$$

$N_{p}$ is the number of POUs, $N_{c}$ is the number of CHP units, and $N_{h}$ is the number of HOUs. The amounts of power generated in terms of MW by *ith* POU and *ith* CHP unit are $P_{i}$ and $O_{i}$, respectively. $H_{i}$ and $T_{i}$ represent the amount of output heat of the *i-th* unit of CHP and HOU in MWth, respectively. The total cost function in Equation (1) consists of the sum of the cost functions of the POUs, CHPs, and HOUs. Also, $C_{pi}\left(P_{i}\right)$, $C_{ci}\left(O_{i},H_{i}\right)$, $C_{hi}\left(T_{i}\right)$ represent the operation costs (all in $/h) of POUs, CHP units, and HOUs, respectively.

The cost function of POUs is shown by considering the VPLE of thermal power units with $C_{pi}$. This term adds a sinusoidal component to the cost function and makes it uneven. Equation (2) presents the cost function of POUs, considering the VPLE constraint, as follows:

(2) $$C_{pi}\left(P_{i}\right)=a_{i}P_{i}^{2}+b_{i}P_{i}+c_{i}+\left|d_{i}Sin\left\{e_{i}\left(P_{i}^{min}-P_{i}\right)\right\}\right|$$

$a_{i}$ ($/MW^2^h), $b_{i}$ ($/MWh), and $c_{i}$ ($/h) are the fuel cost factors of the *ith* POU, $d_{i}$ ($/h) and $e_{i}$ (rad/MW) are the fuel cost factors related to the VPLE of the thermal unit *i*. Also, $P_{i}^{min}$ is the minimum generated power of the thermal unit *i*.

The cost function of cogeneration units is modeled as [83]:

(3) $$C_{ci}\left(O_{i},\;H_{i}\right)=\alpha_{i}O_{i}^{2}{+\beta }_{i}O_{i\;}{+\gamma }_{i\;}{+\delta }_{i}H_{i\;}^{2}{+\varepsilon }_{i}H_{i\;}{+\xi }_{i}O_{i}H_{i}$$

$\alpha_{i}$, $\beta_{i}$, $\gamma_{i}$, $\delta_{i}$, $\varepsilon_{i}$ and $\xi_{i}$ are the cost factors of *i-th* CHP unit and are in $/MW^2^ h, $/MW h, $/h, $/MWth^2^ h, $/MWth h, and $/MW MWth h, respectively.

Furthermore, the fuel cost of the HOU is formulated as [84]:

(4) $$C_{hi}\left(T_{i}\right)=\eta_{i}T_{i}^{2}+\theta_{i}T_{i}+\lambda_{i}$$

where $\eta_{i}$*,* $\theta_{i}$, and $\lambda_{i}$ are the cost factors of the *i-th* boiler and are in terms of $/MWth^2^ h, $/MWth h and $/h, respectively.

### 2.2. Constraints

#### 2.2.1. Equality Constraints

These equalities are as follows:

(5) $$\overset{N_{p}}{\underset{i=1}{\sum }}P_{i}+\overset{N_{c}}{\underset{i=1}{\sum }}O_{i}-P_{d}=0$$

(6) $$\overset{N_{c}}{\underset{i=1}{\sum }}H_{i}+\overset{N_{h}}{\underset{i=1}{\sum }}T_{i}-H_{d}=0$$

$P_{d}$ and $H_{d}$ are the demands for electric and thermal power in terms of *MW* and *MWth*, respectively. Equation (5) describes the equilibrium constraint of active power [35]. This constraint balances the electrical power generated by the POUs and the CHP units with the total electrical power demand of the system. In addition, Equation (6) shows the thermal power constraint [40], which balances the total heat generated by CHP units and HOUs with the system heat demand.

#### 2.2.2. Inequality Constraints

- Capacity limitations of Power-only units

The amount of generated capacity by POUs should be limited within a permissible range and is expressed as:

(7) $$P_{i}^{min}\leq P_{i}\leq P_{i}^{max}$$

$P_{i}^{max}$ and $P_{i}^{max}$ are the upper and lower limits of the *i-th* thermal power plant in the system.

- Permissible limits of boilers

The amount of heat that a boiler unit can produce is in a certain bound, which is modeled as follows:

(8) $$T_{i}^{min}\leq T_{i}\leq T_{i}^{max}$$

$T_{i}^{max}$ and $T_{i}^{max}$ are the upper and lower bounds of the i-th boiler of the system.

- Permissible limits of CHP units

Active power generated by POUs and the heat produced by HOUs is limited by the relevant minimum and maximum limits. Also, the generated heat and power by CHP units are limited to the FOR, described by Equations (9) and (10).

(9) $$O_{i}^{min}\left(H_{i}\right)\leq O_{i}\leq O_{i}^{max}\left(H_{i}\right)$$

(10) $$H_{i}^{min}\left(O_{i}\right)\leq H_{i}\leq H_{i}^{max}\left(O_{i}\right)$$

$O_{i}^{min}$ and $O_{i}^{max}$ are the upper and lower bounds of the generated power by CHP units, respectively, and $H_{i}^{min}$ and $H_{i}^{max}$ are the lowest and highest possible produced heat by the CHP units, respectively.

## 3. The ICHHO Structure

Generally, the hybrid metaheuristics (HMHs) are divided into low, and high levels. In low-level HMHs, a given function of one metaheuristic method is replaced by another one, and there is no direct relationship to the internal working of a metaheuristic at the high level. All HMHs in both low, and high levels are implemented by relay, or parallel processing. In relay hybridization, a set of metaheuristics are applied one after another, while parallel type provides cooperative optimization models. In general view all of the HMHs, can be classified into homogeneous, or heterogeneous; global, or partial; and general, or specialist [85].

The ICHHO, is a type of parallel, high-level and heterogeneous hybrid algorithms with partial search. The ICHHO algorithm can pursue several optimum solutions using the multi-swarm structure of the ICA, and its revolution mechanism to diversify solutions. Also, the time-varying randomized nature of HHO, due to parameters of Levy Flight-based search, and escaping energy patterns, helps the ICHHO avoid being trapped in local minimums [86].

In the proposed ICHHO, for the combination of ICA and HHO algorithms, the ICA algorithm is referred to as the base algorithm. The ICA has an operator called assimilation operator, which is removed in ICHHO and replaced with HHO algorithm instead.

The ICHHO, was firstly introduced in [86], as a combination of the ICA with the HHO. The superiority of the ICHHO algorithm in solving different mathematical benchmark functions, include unimodal, and multimodal optimization problems were completely proved over GA, PSO, ICA, and HHO, in terms of the average (Ave), minimum (min), and the standard deviation (SD) of obtained results [86].

The HHO was initially introduced in 2019 [87], concludes with three main stages an exploration phase, exploitation phase, and transition between these two phases. Interested readers are referred to [81] for further descriptions.

The ICA is adapted from the evolution process of global communities, and it is very popular due to its high speed and accuracy in finding solutions to optimization problems [88]. In ICA, several initial populations are randomly generated. Each member of the population is called a “country”. An arbitrary number of the most powerful countries are considered colonizers and the rest colonies. In ICA, each country is a solution to the optimization problem.

The main weakness of the HHO algorithm is the exploration phase, which is due to the weakness of its search mechanism, in such a way that in the HHO algorithm, the parameter *E* (escaping energy of the rabbit) is a variable to determine the phase of the algorithm, exploration or exploitation. For this purpose, the following formula is used:

(11) $$E=2E_{0}\left(1-\frac{t}{T}\;\right)$$

$E_{0}$ is the initial energy of the rabbit, and in each iteration, it is updated in the interval of [−1,1]. *t* and *T* are the iteration number and maximum iteration of the algorithm, respectively. For HHO to be in the exploration phase, *E* must be greater than 1. According to Equation (11), when the algorithm reaches the second half of the iteration (that is, *t* >= *T*/2), the value of *E* cannot be greater than 1, and this means that the search mechanism of the algorithm is lost in all iterations of *t* >= *T*/2. The strength of the HHO algorithm is its exploitation phase, which prevents the algorithm from getting trapped in local optima. This is due to the existence of the levy flight (LF) function in the algorithm. Parameter *E*, which is mainly placed in the exploitation phase, is also effective in this subject. On the other hand, ICA suffers from premature convergence. Because during the colonial competition of the colonizer countries, the number of empires decreases and when the number of empires reaches 1, the new calculations are finished before the algorithm reaches the maximum number of iterations. Therefore, fast convergence occurs and the algorithm gets trapped in local optima.

The literature [86], confirms that the HHO has a weak search mechanism, but it is powerful in the exploitation phase, because of its time-varying nature due to random parameters. These prevent the HHO algorithm to trap in local optima. On the other hand, ICA, has a powerful search mechanism, which diversifies solutions. The hybrid ICHHO algorithm performs well, and the above features ensure that this algorithm is protected from premature convergence and entanglement in local optima. In addition, the mechanism of the ICHHO algorithm has been fully and comprehensively explained in the authors’ previous research (ref. [81]). For more details, the interested readers are referred to mentioned reference, to avoid repetition.

### 3.1. The Flowchart of the ICHHO Algorithm

**Figure 1:**
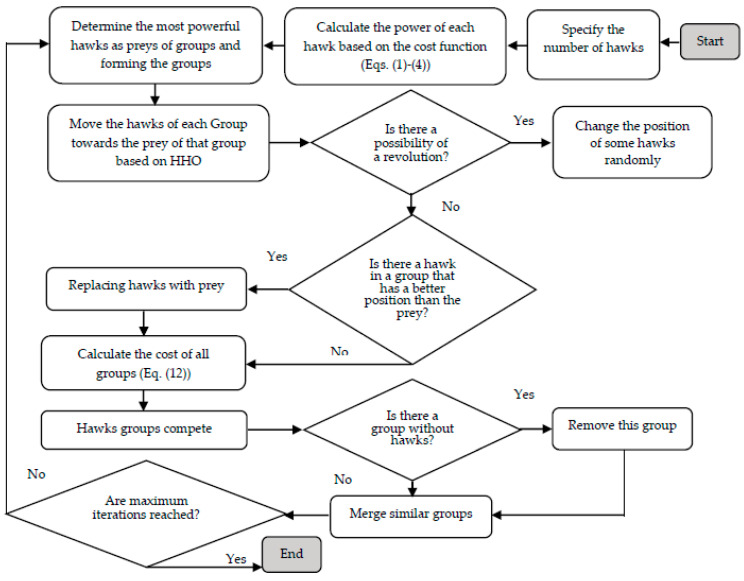
The general flowchart of the ICHHO algorithm.

Figure 1 shows the flowchart of the ICHHO hybrid algorithm.

The general steps of the ICHHO to solve the optimization problems, are explained briefly below.

Step 1. Data entry.

Step 2. Generating the hawks randomly.

Step 3. Moving all the hawks of all groups in the direction of the rabbit of their group.

Step 4. Applying the revolution factor.

Step 5. Shifting and moving the to the rabbit, based on the specified strategy.

Step 6. Calculation of the cost of all groups, as:

(12) $$TC_{i}=f_{i}^{bait}+\xi .mean\left(f_{i}^{Hawks}\right)$$

where $TC_{i}$ is the total cost of *i-th* group, and ξ is a number between zero and one.

Step 7. Electing several hawks from the groups with the lowest power.

Step 8. Eliminating the weak groups.

Step 9. Competition.

Step 10. Stop, and print the results.

### 3.2. The Main Steps of Solving the CHPED Problem by the Proposed Algorithm

Here, the main steps for applying the ICHHO algorithm to the CHPED problem are described.

It should be noted that in the CHPED problem, the decision variables to be optimized are the output powers of POUs, and CHP units (i.e., $P_{i}$ and $O_{i}$, respectively), and the output heats of HOUs, and CHP units (i.e., $H_{i}$ and $T_{i}$, respectively).

Step 1. Setting algorithm parameters: These parameters are the number of hawks (*NHawks*), number of groups (*NGroups*), number of algorithm iterations (*T*), revolution probability, and ξ. *NHawks*, *NGroups*, and *T* are considered different values for different test systems, and their values are specified in related simulation sections. Also, the number of groups, revolution probability, and ξ for all test systems are equal to 10, 0.6, and 0.2, respectively.

Step 2. Determining the primary position of hawks; the power and heat produced by the units are the variables of the problem, which are supposed as the hawks in ICHHO. Each hawk is a vector that includes the powers of POUs and CHP units, as well as the heat of CHP units and boilers. Therefore, every hawk is a solution to the CHPED problem. The positions of the hawks are generated through Equations (13) to (16) [45], to satisfy the inequality constraints of the problem, as:

(13) $$P_{ri}=P_{ri}^{min}+rand\times \left(P_{ri}^{max}-P_{ri}^{min}\right),\;\;r=1,\ldots N_{p}$$

(14) $$O_{si}=O_{si}^{min}+rand\times \left(O_{si}^{max}-O_{si}^{min}\right),\;\;s=1,\ldots N_{c}$$

(15) $$H_{ti}=H_{ti}^{min}+rand\times \left(H_{ti}^{max}-H_{ti}^{min}\right),\;\;t=1,\ldots N_{c}$$

(16) $$T_{ui}=T_{ui}^{min}+rand\times \left(T_{ui}^{max}-T_{ui}^{min}\right),\;\;u=1,\ldots N_{h}$$

Step 3. Calculation of the cost of all hawks; In step 2, the hawks position was generated randomly. Here, the cost of each hawk is calculated. To satisfy the equality constraints, two methods have been used consecutively. First, the method of correcting the answers is used. In this way, the values of $P_{error}$ and $H_{error}$ are determined as follows:

(17) $$P_{error}=P_{d}-\left(sum\left(P_{k}\right)+sum\left(O_{k}\right)\right)$$

(18) $$H_{error}=H_{d}-\left(sum\left(T_{k}\right)+sum\left(H_{k}\right)\right)$$

Here, $sum\left(P_{k}\right)$ and $sum\left(O_{k}\right)$ are the summations of produced power by entire POUs and CHP units, respectively. Likewise, $sum\left(T_{k}\right)$ and $sum\left(H_{k}\right)$ are summations of produced heat by entire CHP units and HOUs.

To accurately match the total amounts of power and heat generation with consumption values and not to violate these very important constraints, the power and heat production values are changed until $P_{error}$ and $H_{error}$ are equal to zero, and the iteration process continues. This issue will ensure complete and accurate compliance with the equality constraints of production with the consumption of power and heat generation values. In addition to this method, to fully ensure the satisfaction of the equality constraints, the penalty function method has been used by assuming a weight of 10^6^.

Step 4. Determining the rabbit and groups.

Step 5. Moving the hawks of any group toward its rabbit.

Step 6. Changing the hawk position by using the revolutionary operator if possible.

Step 7. Replacing a hawk with a rabbit in the group, if that was better.

Step 8. Updating the cost of hawks and rabbits. Then between the groups, colonial competition is applied. During this competition, if a group is without a hawk, it will be eliminated.

Step 9. Repeat steps 5 to 8 until reaching maximum iterations. Otherwise, go to stage 10.

Step 10. Print the position and cost of the strongest group’s rabbit as the best solution found by the algorithm.

### 3.3. The Complexity of the ICHHO Algorithm

The computational complexity of metaheuristic algorithms is depending on the numbers of steps they call cost function, which is shown by O(). By assuming the *T*, *N*, and *D* as the number of iterations, the size of the population, and dimensional size of the problem, the upper, and lower bounds of complexity for HHO are O(N×T×D+0.75×N×T×D), and O(N×T×D). It should be noted that in the HHO algorithm, it is assumed that a maximum of 75% of all hawks will participate in update phase of positions. This imposes 0.75×N×T×D further computational complexity. The upper, and lower bounds for ICA, are O(N×T×D+(N−I)×T×D), and O(N×T×D), respectively, where I is the number of imperialists. Considering that the ICHHO is established on the ICA framework, the lower, and upper bounds of ICHHO are estimated as O(N×T×D), and O(1.75×N×T×D+(N−I)×T×D), respectively [88,89].

## 4. Simulation Results

In this section, the efficiency of the ICHHO algorithm to solve the CHPED problem in four different case studies is analyzed. In all test systems, the VPLE of POUs is considered. Also, the quality of the solutions in terms of the operation cost value obtained for each test system by the proposed algorithm is compared with the other algorithms in this field. It should be noted that for some algorithms in the literature, the actual value of objective function is calculated according to the generation values of the units, which have been addressed in that reference. This may be different from the results reported for the cost function of that reference.

To make a fair comparison between the results of the proposed algorithm and other algorithms, for each test system, the number of iterations was exactly selected equal to the what considered in previous researches. Therefore, the number of iterations for test systems 1, and 2 (24, and 48 units) is assumed to be 500 [20,37,41,45,46,70,90], and for test systems 3, and (84, and 96 units), it is assumed to be 1000 [30,37,41,62,91]. Also, the size of the population affects the run-time of the algorithm. For each test system, to improve the optimal solutions compared to previous researches, in the form of trial and error the method was run several times with different population sizes (the number of hawks). Finally, a population was allocated that is optimally obtain better solutions than previous researches in reasonable run-time. It means that the population size is equal to 2000 for test system 1 (24-unit), and 300 for test systems 2–4 (48-unit, 84-unit, and 96-unit).

To compare the results of the ICHHO algorithm with other methods (in the test systems of 24 and 48 units), due to the number of used algorithms to solve the mentioned problem, the algorithms that had better results, was selected. For this reason, the compared algorithms for these two test systems are different. Also, the algorithms used for solving the problem in very large-scale systems (84, and 96 units) are completely different with the medium, and large-scale systems. There are only a few algorithms that have been used to solve very large-scale systems. Consequently, different algorithms were inevitably used for comparative studies. It should be noted that the ξ is selected 0.2 for all test systems. The detailed data of all case studies are presented in Appendix A.

### 4.1. Test System 1

The first test system is a medium-scale system with 24 units, including 13 POUs, 6 CHP units, and 5 HOUs. The power and heat demand of this system is 2350 MW and 1250 MWth, respectively. The data of this test system is extracted from the ref. [45]. The results obtained from the ICHHO algorithm are presented in Table 2, and compared with the GSA [45], HBOA [41], IGSO [20], ICA, and MICA [90] algorithms. However, it should not be forgotten that for all reported algorithms, one, or both of the power/heat balances are violated and they are not fully satisfied. This raises serious doubt about the better results obtained by those algorithms in terms of mean and standard deviation than the ICHHO. This unacceptability is much more fundamental in the case of the results of some algorithms in which the constraints on power or heat generation are violated. So, it can be concluded that the results obtained by the ICHHO algorithm, in this case, are certainly better than the other reported results.

**Figure 2:**
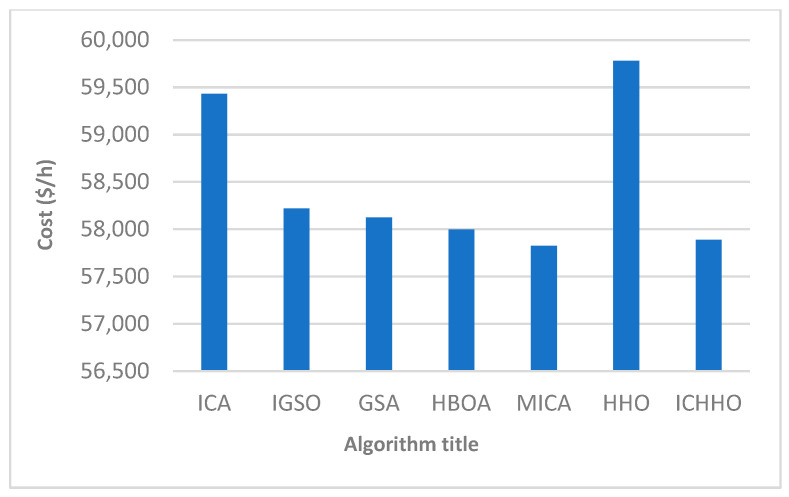
Comparison of obtained operating costs by different algorithms in a 24-unit system. In MICA [90], the power, and heat balances were violated equal to −0.0009, and are not acceptable, compared to the ICHHO algorithm.

**Figure 3:**
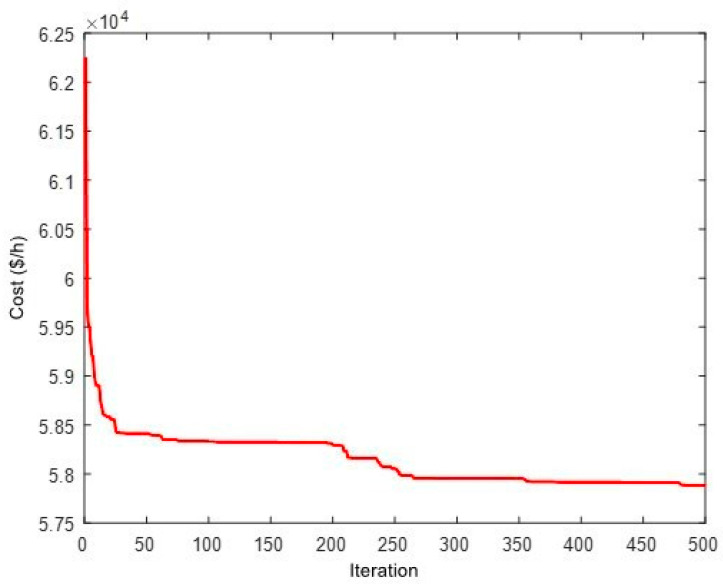
Convergence curve of ICHHO for 24-unit test system.

The total fuel costs obtained by different algorithms are depicted in Figure 2. The results show that the ICHHO algorithm performs better than other methods, in terms of lower cost. For example, assuming a constant annual load, the ICHHO algorithm decreases the annual operating cost by about $950,109 and $13,540,857 compared to HBOA [41], and ICA [90], respectively. Also, the comparative results confirm that, the total costs in the ICHHO algorithm are reduced by about 0.4057%, 0.1870%, 0.5721%, and 2.6008% compared to GSA [45], HBOA [41], IGSO [20], and ICA [90], respectively. Since the heat and power balances are violated with the application of MICA [90], they may not be compared in this case with the performance of the proposed ICHHO algorithm, where there is no violation balance is equal to 0.0000 (see Table 2). Furthermore, the convergence curve of the ICHHO algorithm is shown in Figure 3. The interested readers are referred to references [20,41,45], and [90] to analyze the convergence curve of the other algorithms.

The CHPED problem has already been solved and investigated with the ICA algorithm, and its variant versions, whose solutions, and our detailed investigations for some cases are addressed based on ref [90]. Regarding the HHO algorithm, its solutions for the studied problem are very weak, have very high operating costs, and it cannot compare and compete with other algorithms. As an example, for the first test system, the results of applying the ICA, and HHO, were presented, separately which confirm the inability of these algorithms compared to the combined version. To summarize, for other studied systems (except for the second system for ICA), the results of the above algorithms have been avoided.

### 4.2. Test System 2

**Figure 4:**
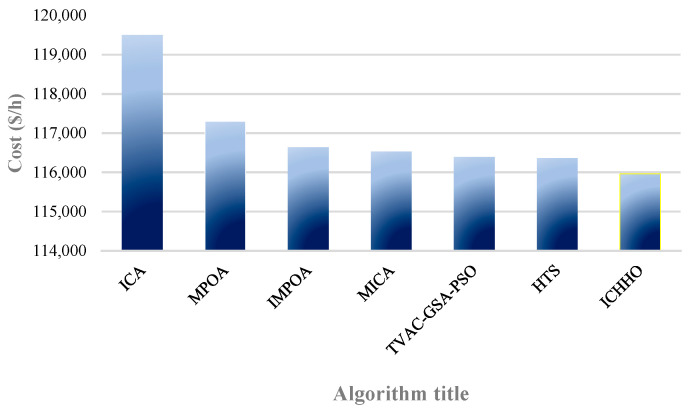
Comparison of operating costs obtained by different algorithms for test system 2.

**Figure 5:**
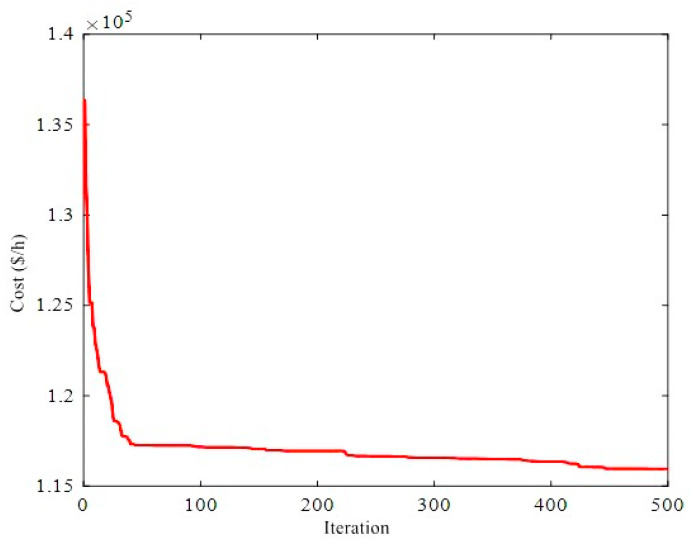
Convergence curve for test system 2.

Test case 2 is a 48-unit large-scale system with 26 POUs, 12 CHP units, and 10 HOUs. The electric and thermal loads of this system are 4700 MW, and 2500 MWth. The data of this system is extracted from [45]. Table 3 compares the results of the proposed algorithm and the operation costs obtained by the algorithms, including TVAC-GSA-PSO [70], MPOA and IMPOA [37], HTS [46], ICA, and MICA [90]. The results show the superiority of the ICHHO performance over the reported algorithms. The comparative results confirm that, the total costs in ICHHO algorithm are reduced by about 0.3684%, 1.1448%, 0.5796%, 0.342%, 2.9580%, and 0.4859% compared to TVAC-GSA-PSO [70], MPOA [37], IMPOA [37], HTS [46], ICA [90], and MICA [90] respectively. Furthermore, the annual operating savings of the proposed method compared to IMPOA, and MICA is about $5,922,254, and $4,960,941, respectively. Figure 4 shows the cost of operation by different algorithms for a 48-units system. The ICHHO convergence curve for test system 2 is shown in Figure 5.

### 4.3. Test System 3

**Figure 6:**
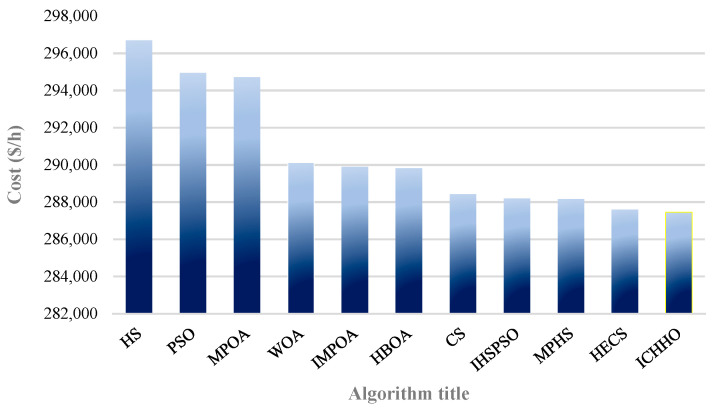
The comparison of the costs for different algorithms for an 84-unit system.

**Figure 7:**
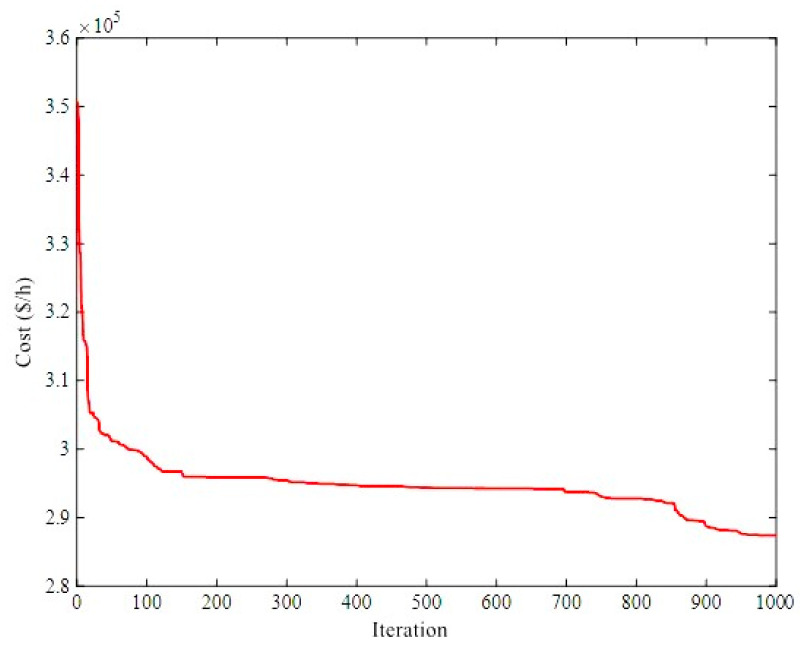
The convergence curve of the ICHHO algorithm for test system 3.

This case, as a large-scale system, consists of 84 units; 40 POUs, 24 CHP units, and 20 HOUs. The power and heat demands are 12,700 MW and 5000 MWth, respectively. The data of this system is extracted from Ref. [30]. Table 4 addresses the generated power and heat by applying the ICHHO algorithm. Also, Table 5, provides the total operating costs compared with the other algorithms. It should be noted that the proposed ICHHO-based CHPED problem is developed and programmed in Matlab R2019b environment and implemented on an Intel(R) Core(TM) i5-6200U CPU @ 2.30 GHz, 2.40 GHz, 4 GB RAM, 64-bit operating system, x64-based processor PC (Acer, Aspire E5-575 series, N16Q2, 2016, China). The results confirm decreasing the total cost of the ICHHO algorithm compared to other algorithms in the range of 0.052241–3.116385% (HECS [91], HS [62] respectively). Figure 6, also provides a comparison of total annual operating costs by different algorithms, assuming the constant load curves over the year. The results verify that the performance of the proposed algorithm is superior to other algorithms. For example, ICHHO’s operational cost savings over HBOA is $20,775,730.2. Figure 7 shows the convergence curve obtained from the proposed method on an 84-unit system.

### 4.4. Test System 4

**Figure 8:**
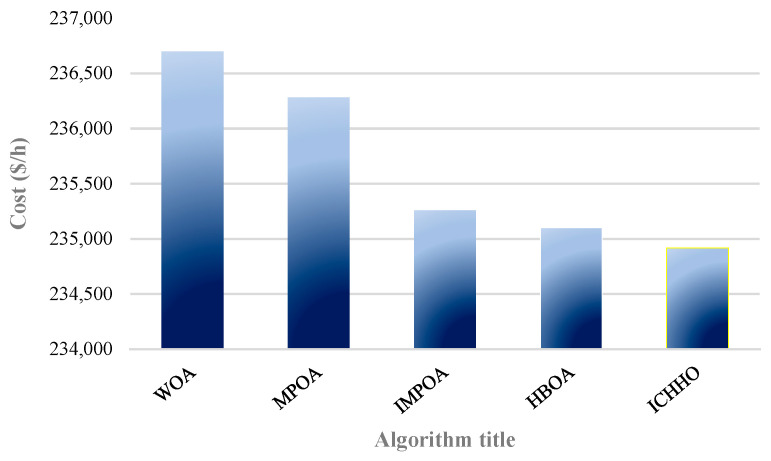
The comparison of the operating costs by different algorithms for the system 4.

**Figure 9:**
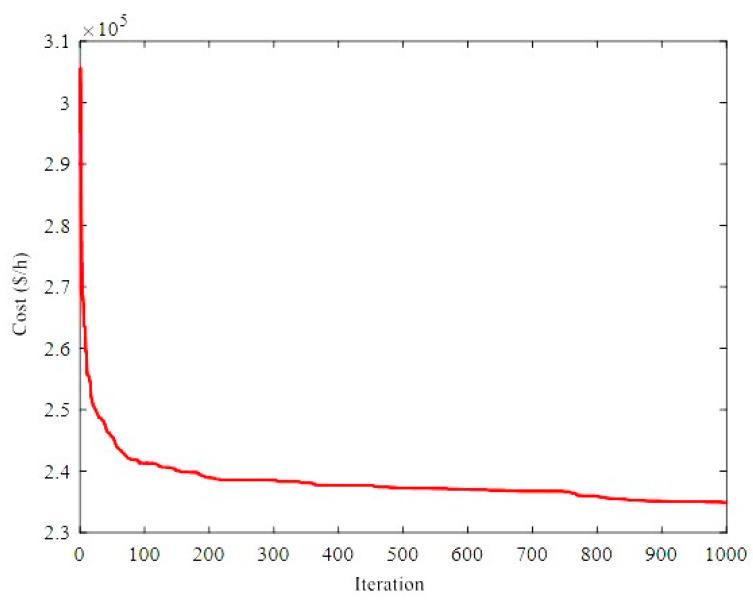
The convergence curve of the ICHHO algorithm for test system 4.

This very large-scale system consists of 96 units, including 52 POUs, 24 CHP units, and 20 HOUs. The power demand is 9700 MW and the required heat is 5000 MWth. The data of this system are available in Ref. [30]. Table 6 presents the output power or heat obtained by ICHHO and Table 7 compares the operating costs of the ICHHO algorithm with other algorithms, including WOA [30], HBOA [41], MPOA [37], and IMPOA [37]. The results confirm decreasing the total cost of the ICHHO algorithm compared to other algorithms in the range of 0.07875–0.75271% (HBOA [41], WOA [30] respectively). The results show a better performance of the ICHHO than different algorithms. The annual capital savings compared to WOA, HBOA, MPOA, and IMPOA are $15,607,353.8, 1,622,012.9, 11,962,754.9, and 3,003,026.9, respectively. Figure 8 shows a comparison of the costs of different algorithms. The ICHHO convergence curve for system four is shown in Figure 9.

### 4.5. Sensitivity Analysis for 24-Unit System

In this section, a simple sensitivity analysis is presented to describe the dependence of the final solutions on the setting parameters. For this purpose, in the 24-unit system (Test System 1), the effect of the parameters on the final solutions is evaluated in three different cases. In the first one, the revolution probability, and ξ are selected equal to 0.6, and 0.2 (the relevant results are depicted in Table 2), respectively. Then these parameters are changed to 0.5, and 0.2; and finally selected as 0.4, and 0.1, respectively. The obtained results are depicted in Table 8.

Table 8 confirms that by changing the setting parameters of the algorithm, the total cost of the problem, will be increased seriously. This clarifies the significant impacts of optimal parameter setting on final results.

### 4.6. Main Findings

The purpose of this work is to investigate the performance of the ICHHO algorithm to obtain better solutions to the CHPED problem. In the simulated case studies, none of the problem constraints are violated (see for example Table 2, the power, and heat balances are equal to zero), and the obtained solutions are better than the other reported techniques. We should take in mind that for some of the reported algorithms, as indicated in Table 2 the power or heat balance constraints are not satisfied. This raises some doubt about the better results reported by those techniques, compared to the ICHHO algorithm. In Section 4.1, Section 4.2, Section 4.3 and Section 4.4, four case studies are analyzed to confirm the performance of the presented algorithm in solving complex power system problems.

- One of the most significant subjects that should be mentioned by the researchers is the validation of the results reported by different references, which can be easily checked by analyzing the total operation costs based on reported power and heat values
- In test system 1, a 24-unit system, as a medium-scale system is simulated and the results confirmed superior of the ICHHO algorithm in finding total cost, compared to other techniques in the range of 0.1870% to 2.6008%.
- In test system 2, a 48-unit system, as a large-scale system, is simulated and the results proved superior of the ICHHO algorithm in finding total cost, compared to other methods in the range of 0.342% to 2.9580%.
- In test system 3, an 84-unit system, as a large-scale system, is simulated and the results verified superior of the ICHHO algorithm in finding total cost, compared to other algorithms in the range of 0.052241–3.116385%.
- In test system 4, a 96-unit system, as a very large-scale system, is simulated and the results confirmed the superiority of the ICHHO algorithm in finding total cost, compared to other techniques in the range of 0.07875–0.75271%.
- Similar to all comparative studies conducted in previously published research in this field; the improvement of the results of the studied problem by applying the proposed algorithm is very low (less than half a percent). It should be noted that the results obtained are based on the standard period of defining the CHPED problem of one hour, which by assuming the constant power and heat profile in a one year, the amount of cost saved will be very significant, and in the range of thousands, or even millions of dollars. Based on this, the cost reductions by the application of the proposed algorithm compared to the best solutions in the literature are equal to $852,173; $270,714; $1,864,543; and $1,622,013 for the four studied systems, respectively.
- Finally, the proposed algorithm is a combination of two strong meta-heuristic algorithms that simultaneously take the advantage of the good features of the two algorithms. It is a strong algorithm in terms of speed and has an acceptable mechanism in terms of changing from the exploration phase to the exploitation and vice versa. On the other hand, this algorithm can find the optimal points of the CHPED problem, as one of the most complex and non-linear problems in power system engineering. Therefore, it can be claimed that the ICHHO algorithm can be applied to other engineering optimization problems.

## 5. Conclusions

The CHPED problem is an essential concept in power system operation studies, aiming to minimize the total cost of generation, while satisfying different types of constraints and limitations. In this paper, a short review of applied algorithms to handle the CHPED problem in three main categories namely classical, or conventional methods, stochastic search-based techniques (evolutionary or heuristic algorithms), and hybrid approaches are presented. Also, some details on problem constraints, and different case studies classified in the small, medium, large, and very large scales are provided. Then, the combined ICHHO algorithm, as a combination of ICA and HHO algorithms, is applied to the CHPED problem. The mentioned problem is solved considering the VPLE of the POUs, and the generation limits of POUs, HOUs, and CHP units. In addition, the interdependence of heat and power in CHP units, which causes the complexity of the problem is modeled. As shown, ICHHO can find better solutions to the CHPED problem in different case studies of 24-unit as a medium-scale system, 48-unit, and 84-unit as large-scale systems, and 96-unit as a very large-scale system. Specifically, in the 24-unit test system, the amount of reduction in operating costs using the ICHHO algorithm compared to the GSA, HBOA, IGSO, and ICA algorithms is 0.4057%, 0.187%, 0.5721%, and 2.6008%, respectively. It saves the amounts of $235.81, $108.46, $333.09, and $1545.76 per hour. In the 48-unit test system, the cost reduction percentages of ICHHO compared to TVAC-GSA-PSO, MPOA, IMPOA, HTS, ICA, and MICA algorithms are 0.3684% ($428.8599/hour saving), 1.1448% ($1342.9565/hour saving), 0.5796% ($676.0565/hour saving), 0.342% ($397.9565/hour saving), 2.958% ($3534.8006/hour saving), and 0.4859% ($566.3175/hour saving), respectively. In the large test system of 84 units, the ICHHO algorithm causes lower operation costs compared to the HECS, MPHS, IHSPSO and CS algorithms, equal to $150.2452 per hour (−0.0522%), $706.6984 per hour (−0.2452%), $745.8681 per hour (−0.2588%), and $967.9478 per hour (−0.3356%). In the very large test system of 96 units, the results verify the lower operation costs of ICHHO algorithm compare to the HBOA, IMPOA, MPOA and WOA algorithms equal to: $185.1613 per hour (−0.07875%), $342.8113/hour (−0.14571%), $1365.6113/hour (−0.57795%), and $1781.6614/hour (−0.75271%). By examining the run-time of the ICHHO algorithm for large-, and very large-scale systems, it is evident that the proposed algorithm has a suitable and acceptable performance. As a suggestion for future research, the above algorithm can be applied to the multi-objective CHPED problem. Also, to bring the situation closer to reality, other practical constraints, such as prohibited operation zones of POUs, and the impacts of different uncertainties can be included.

## Figures and Tables

**Table 1:** A taxonomy of the proposed algorithms to solve the CHPED problem in terms of constraints, and case studies.

Ref.	Algorithm	Constraints				Case Study (s)	
		VPLE	POZ	RRL	TLs	System Scale	Number of Units
[7]	MEMA	-	-	-	-	Small	5
[2]	EMA	● *	-	-	●	Small, Medium, Large	5, 7, 24, 48
[5]	BLPSO	●	●	-	●	Small, Medium, Large	5, 7, 24, 48
[6]	GOA	-	-	-	-	Small	5
[39]	TSCO	●	-	-	●	Small	5, 7
[11]	LR	-	-	-	-	Small	4
[12]	Two-layer LR	-	-	-	-	Small	4
[21]	DE	●	-	-	-	Small	7
[22]	EP	-	-	-	-	Small	4
[28]	BCO	●	-	-	●	Small	7
[44]	HSA	●	-	-	-	Medium, Large	24, 84
[79]	HS	-	-	-	-	Small	4, 5
[80]	HS	-	-	-	-	Small	5
[24]	GA	-	-	-	-	Small	4
[45]	GSA	●	-	-	●	Small, Medium, Large	5, 7, 24, 48
[30]	WOA	●	-	-	-	Medium, Large, Very large	24, 84, 96
[31]	COA					Medium, Large	24, 48
[40]	ICA	●	-	-	●	Small, Medium, Large	5, 7, 24, 48
[32]	GSO	●	●	-	●	Small, Medium, Large	4, 7, 24, 48
[41]	HBO					Medium, Large, Very large	24, 84, 96
[36]	IWO	-	-	-	-	Small	4, 5
[38]	ABC	●	-	-	●	Small	7
[43]	KKO	●	-	-	●	Small, Medium, Large	4, 5, 7, 24, 48
[48]	TVAC-PSO	●	-	-	●	Small, Medium, Large	4, 5, 7, 24, 48
[50]	SPSO	-	-	-	-	Small	4
[52]	RCGA-IMM	●	-	-	●	Small, Medium	4, 5, 7, 24
[53]	IGA-NCM	●	●	-	●	Small, Medium, Large	4, 5, 7, 24, 48
[56]	SGWO	●	●		●	Medium, Large	24, 48
[57]	PFCOA	-	-	-	-	Small	4
[58]	ECSA	●	-	-	●	Small, Medium, Large	4, 5, 7, 11, 24, 48
[62]	IHSPSO	●	-	-	-	Large	84
[81]	ICHHO	●	-	-	●	Small, Large	5, 7, 48
[72]	CSA-BA-ABC	●	●	-	●	Small, Medium, Large	7, 24, 48
This work	ICHHO	●	-	-	-	Medium, Large, Very large	24, 48, 84, 96

* Means this constraint is modeled in the formulation of the problem.

**Table 2:** The obtained results for test System 1 *^,^**.

Output (MW or MWth)	GSA [45]	HBOA [41]	IGSO [20]	ICA [90]	MICA [90]	HHO	ICHHO
P_1_	538.5150	538.5587	628.1520	628.3188	628.3185	363.7601	628.3185
P_2_	224.4727	300.2175	299.74778	63.1622	299.1955	341.6413	298.5145
P_3_	224.4611	301.0825	154.5535	0.0014	299.1614	204.4760	298.8880
P_4_	109.8666	159.7779	60.8460	179.9162	109.8665	103.9807	60.0000
P_5_	109.8666	63.2173	103.8538	179.9147	109.8665	111.1527	60.0000
P_6_	109.9000	60.6889	110.0552	179.9162	109.8662	71.9573	159.1177
P_7_	109.8666	160.2065	159.0773	179.9162	59.9999	156.5307	110.2897
P_8_	109.8666	111.5383	109.8258	179.9162	109.8658	144.5830	159.0836
P_9_	109.8666	161.2539	159.9920	179.9162	109.8572	130.4270	60.0000
P_10_	77.5210	40.0000	41.1030	39.9999	39.9928	48.9790	40.0000
P_11_	77.5341	40.0002	77.7055	50.0917	77.0322	99.3330	76.6974
P_12_	120.0000	55.6579	94.9768	55.0004	54.9986	110.6079	55.0000
P_13_	120.0000	55.2845	55.7143	55.0005	54.9932	102.2519	55.0000
O_1_	92.5632	87.9441	83.9536	117.4866	81.0122	87.7959	81.0008
O_2_	40.0050	41.2662	40.0000	45.9255	39.9995	56.7678	40.0397
O_3_	84.4916	84.0348	85.7133	117.4848	80.9924	110.1992	83.0469
O_4_	40.0079	43.1436	40.0000	45.9155	40.0010	41.4207	40.0017
O_5_	10.0000	11.0824	10.0000	9.9991	10.0013	16.2520	10.0000
O_6_	41.1998	35.0440	35.0000	42.1170	34.9784	47.8840	35.0014
H_1_	111.2790	108.6973	106.4569	125.2766	104.8124	108.3950	104.8004
H_2_	74.9980	76.0927	74.9980	80.1160	75.0082	89.0280	75.0343
H_3_	106.7495	106.4762	107.4073	125.2754	104.8013	120.5814	105.9487
H_4_	74.9978	77.7146	74.9980	80.1074	75.0094	76.1155	75.0014
H_5_	40.0000	40.4643	40.0000	39.9993	40.0048	42.5412	40.0000
H_6_	22.8181	20.0204	20.0000	23.2354	19.9947	18.9431	20.0007
T_1_	458.8811	460.5378	466.2575	415.9857	470.3287	434.3958	469.2145
T_2_	60.0000	60.0000	60.0000	60.0009	60.0099	60.0000	60.0000
T_3_	60.0000	60.0000	60.0000	60.0009	60.0099	60.0000	60.0000
T_4_	120.0000	119.9964	120.0000	120.0009	120.0099	120.0000	120.0000
T_5_	120.0000	120.0000	119.8823	120.0009	120.0099	120.0000	120.0000
Power balance violation (MW)	0.0044	−0.0008	0.26988	−0.0009	−0.0009	0.0000	−0.0001
Heat balance violation (MWth)	−0.2765	−0.0003	0	−0.0006	−0.0009	0.0000	0.0000
Total Cost ($/h)	58,121.86	57,994.51	58,219.14	59,431.81	57,823.14	59,779.68	57,886.05

* For more details on the validity of the reported results, please study Section 4.1. ** There is no meaningful compression between the ICHHO algorithm, and MICA [90], in which the power, and heat balances were violated equal to −0.0009, and are not acceptable, compared to the ICHHO algorithm.

**Table 3:** The obtained results for test system 2.

Output (MW or MWth)	TVAC-GSA-PSO [70]	MPOA [37]	IMPOA [37]	HTS [46]	ICA [90]	MICA [90]	ICHHO
P_1_	448.9221	269.2790	538.5531	538.5705	538.5665	628.3185	628.3186
P_2_	149.6625	299.1629	299.2148	224.5205	76.4028	225.3250	296.6487
P_3_	299.1966	299.0890	299.2003	229.6394	68.6531	224.7586	299.9056
P_4_	109.6519	109.8115	60.3754	159.8146	135.5216	159.7814	159.8846
P_5_	110.6110	109.8657	60.8181	60.0409	161.9568	109.9063	60.0077
P_6_	60.0001	159.7307	109.9287	159.7333	145.3224	159.7354	60.0343
P_7_	160.1395	159.7357	60.1741	159.7483	120.3936	109.8683	110.0751
P_8_	60.0000	159.5370	159.8010	60.3910	147.8076	109.8734	159.8230
P_9_	161.0290	159.7325	109.8948	159.7346	135.9726	159.7348	60.0115
P_10_	114.4208	44.4908	40.0189	77.8308	112.0880	40.9267	40.1064
P_11_	77.3833	114.6860	40.1064	77.4274	108.2171	41.1113	110.9429
P_12_	92.5877	96.3800	92.3945	92.4412	74.2195	55.1796	55.0028
P_13_	92.8866	61.9389	55.1275	55.0051	65.2589	92.4469	55.0590
P_14_	360.0419	269.2699	628.3177	628.3214	248.0558	448.7989	629.4041
P_15_	224.4879	299.1850	299.2274	149.6676	299.2280	225.5280	300.1314
P_16_	359.9566	218.2498	359.9998	224.2057	299.6861	75.5055	299.5513
P_17_	159.7553	109.8680	109.8665	159.8062	142.5869	160.1166	119.9805
P_18_	60.0056	159.7241	60.2765	60.2947	138.8223	110.1600	60.0027
P_19_	159.7582	159.7379	102.5140	109.8813	141.4212	159.7385	159.9499
P_20_	159.6526	159.4922	62.2646	109.9534	142.9812	159.7790	109.9471
P_21_	160.9552	159.7297	110.4417	109.8681	119.5467	159.7420	60.0144
P_22_	160.1864	109.8668	159.7715	159.7347	139.5290	160.1720	60.0392
P_23_	77.5641	40.4500	45.5835	77.4085	77.8037	40.2144	40.0214
P_24_	40.0019	88.4952	40.7168	77.7089	81.7250	40.3064	40.0217
P_25_	92.4140	55.9886	55.0251	92.4043	110.3924	92.6548	55.0561
P_26_	92.3911	92.1346	55.3902	55.0095	111.6903	92.4681	55.0599
O_1_	117.7621	136.2095	94.3722	87.5554	95.1582	85.9808	88.5069
O_2_	40.0003	44.0936	61.6959	44.0256	54.6874	98.4890	48.4037
O_3_	81.0089	95.6736	91.5150	83.9878	86.2998	81.7305	87.5121
O_4_	50.3820	63.8467	46.2704	44.0149	55.6011	48.9018	40.9270
O_5_	10.0000	24.8498	15.1239	10.0002	10.9034	10.0881	10.0001
O_6_	35.3869	36.3114	35.0208	48.6396	37.9270	39.3100	41.8380
O_7_	86.3778	81.2438	87.9242	86.0222	109.3835	82.0192	81.9815
O_8_	41.1532	42.9237	63.9727	44.0049	61.1959	40.1102	47.0583
O_9_	99.4942	108.5169	93.3873	82.6239	111.9550	81.2957	89.7065
O_10_	79.7715	44.5124	42.7064	50.0926	55.3394	45.6646	40.0429
O_11_	10.0001	21.0887	18.0051	10.0004	22.9130	13.8709	10.0006
O_12_	35.0000	35.0981	35.0027	39.9712	54.7853	30.3870	37.0027
H_1_	125.4197	13507837	112.041	108.4796	108.0122	107.5957	109.0128
H_2_	74.9848	78.5346	93.7297	78.5030	87.6785	125.4914	82.2544
H_3_	104.7938	113.0349	110.7013	106.4777	104.7373	105.2105	108.4546
H_4_	83.9426	95.5863	80.4137	78.4939	88.4668	82.6853	75.8002
H_5_	40.0000	46.3643	42.1962	40.0006	40.3868	40.0381	40.0000
H_6_	20.1537	20.5962	20.0096	26.1998	21.3115	21.9595	23.1082
H_7_	107.8056	104.9370	108.6864	107.6192	120.5256	105.3725	105.3508
H_8_	75.9787	77.5243	95.6950	78.4853	92.0441	75.0960	81.0931
H_9_	115.1684	120.2418	111.7521	105.7117	122.1583	104.9665	109.6860
H_10_	83.4155	78.8961	77.3372	83.7420	88.2426	79.8908	75.0370
H_11_	39.9999	44.7526	43.4298	40.0004	45.4608	41.6593	40.0002
H_12_	20.0000	20.0448	20.0016	22.2596	28.9895	17.9063	20.9194
T_1_	447.0491	406.4930	430.2195	514.5539	417.0202	436.0609	455.0100
T_2_	59.9870	60.0000	59.9998	60.0000	59.5362	60.0009	60.0000
T_3_	60.0000	60.0000	60.0000	60.0000	59.9175	60.0009	60.0000
T_4_	120.0000	119.9997	119.9999	120.0000	119.9926	120.0009	120.0000
T_5_	119.9999	120.0000	119.9996	119.9997	118.4968	120.0009	120.0000
T_6_	441.3055	437.2111	433.5254	389.4737	418.2604	436.0611	454.2732
T_7_	59.9991	60.0000	59.9998	59.9999	59.7099	60.0009	60.0000
T_8_	59.9993	59.9997	59.9999	59.9999	59.9816	60.0009	60.0000
T_9_	119.9977	119.9998	119.9999	120.0000	119.2701	120.0010	120.0000
T_10_	119.9999	119.9999	119.9996	120.0000	119.7994	120.0010	120.0000
Total Cost ($/h)	116,393.4034	117,307.5	116,640.6	116,362.5	119,499.3441	116,530.8610	115,964.5435

**Table 4:** The generated power and heat by applying the proposed algorithm on test system 3.

Output (MW or MWth)	ICHHO	Output (MW or MWth)	ICHHO	Output (MW or MWth)	ICHHO	Output (MW or MWth)	ICHHO
P_1_	114.0000	P_28_	10.0001	O_15_	57.839	H_18_	40.0062
P_2_	114.0000	P_29_	10.0001	O_16_	45.6852	H_19_	39.9998
P_3_	120.0000	P_30_	97.0000	O_17_	10.0036	H_20_	39.9999
P_4_	190.0000	P_31_	190.0000	O_18_	10.0146	H_21_	23.4183
P_5_	97.0000	P_32_	190.0000	O_19_	10.0001	H_22_	29.2766
P_6_	140.0000	P_33_	190.0000	O_20_	10.0001	H_23_	26.8752
P_7_	300.0000	P_34_	182.5253	O_21_	42.5204	H_24_	30.7534
P_8_	30.0000	P_35_	173.9609	O_22_	55.4085	T_1_	415.6868
P_9_	284.5997	P_36_	166.6678	O_23_	50.1254	T_2_	414.0425
P_10_	279.6092	P_37_	91.8199	O_24_	58.6574	T_3_	414.8845
P_11_	243.5623	P_38_	110.0000	H_1_	115.1600	T_4_	414.8096
P_12_	168.8010	P_39_	93.3524	H_2_	118.8684	T_5_	60.0000
P_13_	349.2790	P_40_	511.2798	H_3_	124.2914	T_6_	60.0000
P_14_	484.0386	O_1_	99.4606	H_4_	122.7610	T_7_	60.0000
P_15_	394.2772	O_2_	106.0687	H_5_	77.9404	T_8_	60.0000
P_16_	394.2696	O_3_	115.7321	H_6_	82.4778	T_9_	60.0000
P_17_	489.3233	O_4_	113.0049	H_7_	77.9922	T_10_	60.0000
P_18_	489.2991	O_5_	43.4062	H_8_	88.1626	T_11_	60.0000
P_19_	511.4435	O_6_	48.6624	H_9_	117.9501	T_12_	60.0000
P_20_	511.2826	O_7_	43.4663	H_10_	129.8036	T_13_	120.0000
P_21_	523.2798	O_8_	55.2478	H_11_	119.0062	T_14_	120.0000
P_22_	523.2811	O_9_	104.4323	H_12_	113.9224	T_15_	120.0000
P_23_	523.3114	O_10_	125.5543	H_13_	82.0298	T_16_	120.0000
P_24_	523.2812	O_11_	106.3143	H_14_	89.5724	T_17_	120.0000
P_25_	523.2817	O_12_	97.2553	H_15_	90.3995	T_18_	120.0000
P_26_	523.2898	O_13_	48.1434	H_16_	79.9077	T_19_	120.0000
P_27_	10.0001	O_14_	56.8809	H_17_	40.0015	T_20_	120.0000

**Table 5:** The comparison of operating costs obtained by ICHHO with different algorithms for test system 3.

Algorithm	Best Operating Cost ($/h)	Percent Reduction of Operating Costs (%)	Run-Time (s)
ICHHO	287,450.7313	0.000000	204.33
PSO [62]	294,952.0378	2.543229	27.76
HS [62]	296,696.9518	3.116385	96.85
IHSPSO [62]	288,196.5994	0.258805	76.32
WOA [30]	290,123.9742	0.921414	158.18
MPHS [44]	288,157.4297	0.245247	76.65
HBOA [41]	289,822.3900	0.818315	-
MPOA [37]	294,717.7000	2.465739	-
IMPOA [37]	289,903.8000	0.846166	-
CS [91]	288,418.6791	0.335605	39.00
HECS [91]	287,600.9765	0.052241	21.76

**Table 6:** The generated power and heat by applying the proposed algorithm on test system 4.

Output (MW or MWth)	ICHHO	Output (MW or MWth)	ICHHO	Output (MW or MWth)	ICHHO	Output (MW or MWth)	ICHHO
P_1_	628.3187	P_31_	109.8441	O_9_	92.5723	H_15_	116.4335
P_2_	226.3286	P_32_	60.0003	O_10_	40.7298	H_16_	76.3449
P_3_	222.3215	P_33_	159.7614	O_11_	10.0174	H_17_	40.0029
P_4_	110.1271	P_34_	159.7244	O_12_	37.8010	H_18_	22.7173
P_5_	60.0006	P_35_	162.6272	O_13_	92.4871	H_19_	108.7694
P_6_	159.2105	P_36_	40.0003	O_14_	40.6873	H_20_	82.5454
P_7_	163.7172	P_37_	40.0773	O_15_	101.7299	H_21_	116.9611
P_8_	60.0180	P_38_	94.8478	O_16_	41.5580	H_22_	77.1718
P_9_	159.7553	P_39_	55.8305	O_17_	10.0069	H_23_	40.0052
P_10_	40.0003	P_40_	538.7590	O_18_	40.9780	H_24_	21.6862
P_11_	109.1245	P_41_	299.6293	O_19_	88.0731	T_1_	443.5606
P_12_	55.0002	P_42_	299.2751	O_20_	48.7407	T_2_	59.9987
P_13_	55.1075	P_43_	109.8902	O_21_	102.6700	T_3_	60.0000
P_14_	629.2198	P_44_	159.8404	O_22_	42.5158	T_4_	120.0000
P_15_	219.6027	P_45_	159.8690	O_23_	10.0122	T_5_	120.0000
P_16_	300.1668	P_46_	60.0140	O_24_	38.7096	T_6_	443.7041
P_17_	109.9159	P_47_	60.0002	H_1_	118.4375	T_7_	60.0000
P_18_	60.0260	P_48_	60.0090	H_2_	92.6511	T_8_	60.0000
P_19_	160.1553	P_49_	40.0233	H_3_	104.9834	T_9_	119.9994
P_20_	159.7735	P_50_	40.0099	H_4_	82.7222	T_10_	119.9996
P_21_	159.7735	P_51_	55.0156	H_5_	40.0035	T_11_	444.2785
P_22_	159.7623	P_52_	93.3327	H_6_	20.4442	T_12_	60.0000
P_23_	77.5648	O_1_	105.3008	H_7_	112.6530	T_13_	60.0000
P_24_	77.4067	O_2_	60.4473	H_8_	75.0605	T_14_	119.9994
P_25_	92.5578	O_3_	81.3269	H_9_	111.2943	T_15_	119.9994
P_26_	55.0003	O_4_	48.9456	H_10_	75.6300	T_16_	443.8259
P_27_	628.3930	O_5_	10.0082	H_11_	40.0074	T_17_	59.9987
P_28_	302.0501	O_6_	35.9773	H_12_	21.2732	T_18_	59.9988
P_29_	224.8599	O_7_	94.9933	H_13_	111.2465	T_19_	119.9996
P_30_	60.0002	O_8_	40.0701	H_14_	75.5933	T_20_	119.9995

**Table 7:** The comparison of operating costs obtained by ICHHO with different algorithms for test system 4.

Algorithm	WOA [30]	HBOA [41]	MPOA [37]	IMPOA [37]	ICHHO
Best operating cost ($/h)	236,699.1501	235,102.650	236,283.10	235,260.30	**234,917.4887**
Percent reduction of operating costs (%)	0.75271	0.07875	0.57795	0.14571	0.00000
Run-time (s)	227.3	-	-	-	232.6

**Table 8:** The comparison of the obtained operating costs of test system 1 by ICHHO, for different setting parameters.

Output (MW or MWth)	Revolution Probability = 0.6; ξ= 0.2	Revolution Probability = 0.5; ξ= 0.2	Revolution Probability = 0.4; ξ= 0.1
P_1_	628.3185	168.8908	284.9689
P_2_	298.5145	186.2532	256.2743
P_3_	298.8880	171.5751	286.5973
P_4_	60.0000	159.7332	60.0000
P_5_	60.0000	60.0000	60.0000
P_6_	159.1177	60.0000	60.0000
P_7_	110.2897	60.0000	109.8666
P_8_	159.0836	60.0000	109.8666
P_9_	60.0000	60.0000	60.0000
P_10_	40.0000	40.0000	40.0000
P_11_	76.6974	40.0000	40.0000
P_12_	55.0000	55.0000	55.0000
P_13_	55.0000	92.3999	92.3999
O_1_	81.0008	86.6059	83.4871
O_2_	40.0397	40.3953	43.4876
O_3_	83.0469	82.8511	92.4920
O_4_	40.0017	46.9045	43.0655
O_5_	10.0000	10.0000	10.0000
O_6_	35.0014	36.6662	37.2406
H_1_	104.8004	109.6789	109.1538
H_2_	75.0343	77.6225	79.1193
H_3_	105.9487	108.2543	114.2196
H_4_	75.0014	87.8523	77.7003
H_5_	40.0000	40.0000	40.0000
H_6_	20.0007	21.7691	21.9203
T_1_	469.2145	2159.8872	1673.8676
T_2_	60.0000	60.0000	60.0000
T_3_	60.0000	60.0000	60.0000
T_4_	120.0000	120.0000	120.0000
T_5_	120.0000	120.0000	120.0000
Total Cost ($/h)	57,886.05	58,408.8088	58,360.6215

## Appendix A

### Appendix A.2. 24-Unit Test System Data [45]

**Unit No.**	**Cost Function**	**Capacity or FOR**
1	$C_{p1}\left(P_{1}\right)=0{.00028P}_{1}^{2}+8{.1P}_{1}+550+\left|300\sin\left(0.035\times \left({-P}_{1}\right)\right)\right|$	$0\;\leq {\;P}_{1}\;\leq \;680$
2	$C_{p2}\left(P_{2}\right)=0{.00056P}_{2}^{2}+8{.1P}_{2}+309+\left|200\sin\left(0.042\times \left({-P}_{2}\right)\right)\right|$	$0\;\leq {\;P}_{2}\;\leq \;360$
3	$C_{p3}\left(P_{3}\right)=0{.00056P}_{3}^{2}+8{.1P}_{3}+309+\left|200\sin\left(0.042\times \left({-P}_{3}\right)\right)\right|$	$0\;\leq {\;P}_{3}\;\leq \;360$
4	$C_{P4}\left(P_{4}\right)=0{.00324P}_{4}^{2}+7{.74P}_{4\;}+240+\left|150\sin\left(0.063\times \left({60-P}_{4}\right)\right)\right|$	$0\;\leq {\;P}_{4}\;\leq \;180$
5	$C_{P5}\left(P_{5}\right)=0{.00324P}_{5}^{2}+7{.74P}_{5\;}+240+\left|150\sin\left(0.063\times \left({60-P}_{5}\right)\right)\right|$	$0\;\leq {\;P}_{5}\;\leq \;180$
6	$C_{P6}\left(P_{6}\right)=0{.00324P}_{6}^{2}+7{.74P}_{6\;}+240+\left|150\sin\left(0.063\times \left({60-P}_{6}\right)\right)\right|$	$0\;\leq {\;P}_{6}\;\leq \;180$
7	$C_{P7}\left(P_{7}\right)=0{.00324P}_{7}^{2}+7{.74P}_{7}+240+\left|150\sin\left(0.063\times \left({60-P}_{7}\right)\right)\right|$	$0\;\leq {\;P}_{7}\;\leq \;180$
8	$C_{P8}\left(P_{8}\right)=0{.00324P}_{8}^{2}+7{.74P}_{8\;}+240+\left|150\sin\left(0.063\times \left({60-P}_{8}\right)\right)\right|$	$0\;\leq {\;P}_{8}\;\leq \;180$
9	$C_{P9}\left(P_{9}\right)=0{.00324P}_{9}^{2}+7{.74P}_{9\;}+240+\left|150\sin\left(0.063\times \left({60-P}_{9}\right)\right)\right|$	$0\;\leq {\;P}_{9}\;\leq \;180$
10	$C_{p10}\left(P_{10}\right)=0{.00284P}_{10}^{2}+8{.6P}_{10}+126+\left|100\sin\left(0.084\times \left({40-P}_{10}\right)\right)\right|$	$40\;\leq {\;P}_{10}\;\leq \;120$
11	$C_{p11}\left(P_{11}\right)=0{.00284P}_{11}^{2}+8{.6P}_{11}+126+\left|100\sin\left(0.084\times \left({40-P}_{11}\right)\right)\right|$	$40\;\leq {\;P}_{11}\;\leq \;120$
12	$C_{p12}\left(P_{12}\right)=0{.00284P}_{12}^{2}+8{.6P}_{12}+126+\left|100\sin\left(0.084\times \left({40-P}_{12}\right)\right)\right|$	$55\;\leq {\;P}_{12\;}\leq \;120$
13	$C_{p13}\left(P_{13}\right)=0{.00284P}_{13}^{2}+8{.6P}_{13}+126+\left|100\sin\left(0.084\times \left({40-P}_{13}\right)\right)\right|$	$55\;\leq {\;P}_{13\;}\leq \;120$
14	$C_{c1}\left(O_{1}{,H}_{1}\right)=0{.0345O}_{1}^{2}+14{.5O}_{1}+2650+0{.03H}_{1}^{2}+4{.2H}_{1}+0{.031O}_{1}H_{1}$	CHP type a *
15	$C_{c2}\left(O_{2}{,H}_{2}\right)=0{.0435O}_{2}^{2}{+36O}_{2}+1250+0{.027H}_{2}^{2}+0{.6H}_{2}+0{.011O}_{2}H_{2}$	CHP type b *
16	$C_{c3}\left(O_{3}{,H}_{3}\right)=0{.0345O}_{3}^{2}+14{.5O}_{3}+2650+0{.03H}_{3}^{2}+4{.2H}_{3}+0{.031O}_{3}H_{3}$	CHP type a *
17	$C_{c4}\left(O_{4}{,H}_{4}\right)=0{.0435O}_{4}^{2}{+36O}_{4}+1250+0{.027H}_{4}^{2}+0{.6H}_{4}+0{.011O}_{4}H_{4}$	CHP type b *
18	$C_{c5}\left(O_{5}{,H}_{5}\right)=0{.1035O}_{5}^{2}+34{.5O}_{5}+2650+0{.025H}_{5}^{2}+2{.203H}_{5}+0{.051O}_{5}H_{5}$	CHP type c *
19	$C_{c6}\left(O_{6}{,H}_{6}\right)=0{.072O}_{6}^{2}{+20O}_{6}+1565+0{.02H}_{6}^{2}+2{.34H}_{6}+0{.04O}_{6}H_{6}$	CHP type d *
20	$C_{h1}\left(T_{1}\right)=0{.038T}_{1}^{2}+2{.0109T}_{1}+950$	$0\;\leq {\;T}_{1}\;\leq \;2695.2$
21	$C_{h2}\left(T_{2}\right)=0{.038T}_{2}^{2}+2{.0109T}_{2}+950$	$0\;\leq {\;T}_{2}\;\leq \;60$
22	$C_{h3}\left(T_{3}\right)=0{.038T}_{3}^{2}+2{.0109T}_{3}+950$	$0\;\leq {\;T}_{3}\;\leq \;60$
23	$C_{h4}\left(T_{4}\right)=0{.052T}_{4}^{2}+3{.0651T}_{4}+480$	$0\;\leq {\;T}_{4}\;\leq \;120$
24	$C_{h5}\left(T_{5}\right)=0{.052T}_{5}^{2}+3{.0651T}_{5}+480$	$0\;\leq {\;T}_{5}\;\leq 120$
	* See Appendix A.1.	

### Appendix A.3. 48-Unit Test System Data [45]

**Unit No.**	**Cost Function**	**Capacity or FOR**
1	$C_{p1}\left(P_{1}\right)=0{.00028P}_{1}^{2}+8{.1P}_{1}+550+\left|300\sin\left(0.035\times \left({-P}_{1}\right)\right)\right|$	$0\;\leq {\;P}_{1}\;\leq \;680$
2	$C_{p2}\left(P_{2}\right)=0{.00056P}_{2}^{2}+8{.1P}_{2}+309+\left|200\sin\left(0.042\times \left({-P}_{2}\right)\right)\right|$	$0\;\leq {\;P}_{2}\;\leq \;360$
3	$C_{p3}\left(P_{3}\right)=0{.00056P}_{3}^{2}+8{.1P}_{3}+309+\left|200\sin\left(0.042\times \left({-P}_{3}\right)\right)\right|$	$0\;\leq {\;P}_{3}\;\leq \;360$
4	$C_{P4}\left(P_{4}\right)=0{.00324P}_{4}^{2}+7{.74P}_{4\;}+240+\left|150\sin\left(0.063\times \left({60-P}_{4}\right)\right)\right|$	$0\;\leq {\;P}_{4}\;\leq \;180$
5	$C_{P5}\left(P_{5}\right)=0{.00324P}_{5}^{2}+7{.74P}_{5\;}+240+\left|150\sin\left(0.063\times \left({60-P}_{5}\right)\right)\right|$	$0\;\leq {\;P}_{5}\;\leq \;180$
6	$C_{P6}\left(P_{6}\right)=0{.00324P}_{6}^{2}+7{.74P}_{6\;}+240+\left|150\sin\left(0.063\times \left({60-P}_{6}\right)\right)\right|$	$0\;\leq {\;P}_{6}\;\leq \;180$
7	$C_{P7}\left(P_{7}\right)=0{.00324P}_{7}^{2}+7{.74P}_{7}+240+\left|150\sin\left(0.063\times \left({60-P}_{7}\right)\right)\right|$	$0\;\leq {\;P}_{7}\;\leq \;180$
8	$C_{P8}\left(P_{8}\right)=0{.00324P}_{8}^{2}+7{.74P}_{8\;}+240+\left|150\sin\left(0.063\times \left({60-P}_{8}\right)\right)\right|$	$0\;\leq {\;P}_{8}\;\leq \;180$
9	$C_{P9}\left(P_{9}\right)=0{.00324P}_{9}^{2}+7{.74P}_{9\;}+240+\left|150\sin\left(0.063\times \left({60-P}_{9}\right)\right)\right|$	$0\;\leq {\;P}_{9}\;\leq \;180$
10	$C_{p10}\left(P_{10}\right)=0{.00284P}_{10}^{2}+8{.6P}_{10}+126+\left|100\sin\left(0.084\times \left({40-P}_{10}\right)\right)\right|$	$40\;\leq {\;P}_{10}\;\leq \;120$
11	$C_{p11}\left(P_{11}\right)=0{.00284P}_{11}^{2}+8{.6P}_{11}+126+\left|100\sin\left(0.084\times \left({40-P}_{11}\right)\right)\right|$	$40\;\leq {\;P}_{11}\;\leq \;120$
12	$C_{p12}\left(P_{12}\right)=0{.00284P}_{12}^{2}+8{.6P}_{12}+126+\left|100\sin\left(0.084\times \left({40-P}_{12}\right)\right)\right|$	$55\;\leq {\;P}_{12\;}\leq \;120$
13	$C_{p13}\left(P_{13}\right)=0{.00284P}_{13}^{2}+8{.6P}_{13}+126+\left|100\sin\left(0.084\times \left({40-P}_{13}\right)\right)\right|$	$55\;\leq {\;P}_{13\;}\leq \;120$
14	$C_{p14}\left(P_{14}\right)=0{.00028P}_{14}^{2}+8{.1P}_{14}+550+\left|300\sin\left(0.035\times \left({-P}_{14}\right)\right)\right|$	$0\;\leq {\;P}_{14}\;\leq \;680$
15	$C_{p15}\left(P_{15}\right)=0{.00056P}_{15}^{2}+8{.1P}_{15}+309+\left|200\sin\left(0.042\times \left({-P}_{15}\right)\right)\right|$	$0\;\leq {\;P}_{15}\;\leq \;360$
16	$C_{p16}\left(P_{16}\right)=0{.00056P}_{16}^{2}+8{.1P}_{16}+309+\left|200\sin\left(0.042\times \left({-P}_{16}\right)\right)\right|$	$0\;\leq {\;P}_{16}\;\leq \;360$
17	$C_{P17}\left(P_{17}\right)=0{.00324P}_{17}^{2}+7{.74P}_{17\;}+240+\left|150\sin\left(0.063\times \left({60-P}_{17}\right)\right)\right|$	$0\;\leq {\;P}_{17}\;\leq \;180$
18	$C_{P18}\left(P_{18}\right)=0{.00324P}_{18}^{2}+7{.74P}_{18\;}+240+\left|150\sin\left(0.063\times \left({60-P}_{18}\right)\right)\right|$	$0\;\leq {\;P}_{18}\;\leq \;180$
19	$C_{P19}\left(P_{19}\right)=0{.00324P}_{19}^{2}+7{.74P}_{19\;}+240+\left|150\sin\left(0.063\times \left({60-P}_{19}\right)\right)\right|$	$0\;\leq {\;P}_{19}\;\leq \;180$
20	$C_{P20}\left(P_{20}\right)=0{.00324P}_{20}^{2}+7{.74P}_{20}+240+\left|150\sin\left(0.063\times \left({60-P}_{20}\right)\right)\right|$	$0\;\leq {\;P}_{20}\;\leq \;180$
21	$C_{P21}\left(P_{21}\right)=0{.00324P}_{21}^{2}+7{.74P}_{21\;}+240+\left|150\sin\left(0.063\times \left({60-P}_{21}\right)\right)\right|$	$0\;\leq {\;P}_{21}\;\leq \;180$
22	$C_{P22}\left(P_{22}\right)=0{.00324P}_{22}^{2}+7{.74P}_{22\;}+240+\left|150\sin\left(0.063\times \left({60-P}_{22}\right)\right)\right|$	$0\;\leq {\;P}_{22}\;\leq \;180$
23	$C_{p23}\left(P_{23}\right)=0{.00284P}_{23}^{2}+8{.6P}_{23}+126+\left|100\sin\left(0.084\times \left({40-P}_{23}\right)\right)\right|$	$40\;\leq {\;P}_{23}\;\leq \;120$
24	$C_{p24}\left(P_{24}\right)=0{.00284P}_{24}^{2}+8{.6P}_{24}+126+\left|100\sin\left(0.084\times \left({40-P}_{24}\right)\right)\right|$	$40\;\leq {\;P}_{24}\;\leq \;120$
25	$C_{p25}\left(P_{25}\right)=0{.00284P}_{25}^{2}+8{.6P}_{25}+126+\left|100\sin\left(0.084\times \left({40-P}_{25}\right)\right)\right|$	$55\;\leq {\;P}_{25\;}\leq \;120$
26	$C_{p26}\left(P_{26}\right)=0{.00284P}_{26}^{2}+8{.6P}_{26}+126+\left|100\sin\left(0.084\times \left({40-P}_{26}\right)\right)\right|$	$55\;\leq {\;P}_{26\;}\leq \;120$
27	$C_{c1}\left(O_{1}{,H}_{1}\right)=0{.0345O}_{1}^{2}+14{.5O}_{1}+2650+0{.03H}_{1}^{2}+4{.2H}_{1}+0{.031O}_{1}H_{1}$	CHP type a *
28	$C_{c2}\left(O_{2}{,H}_{2}\right)=0{.0435O}_{2}^{2}{+36O}_{2}+1250+0{.027H}_{2}^{2}+0{.6H}_{2}+0{.011O}_{2}H_{2}$	CHP type b *
29	$C_{c3}\left(O_{3}{,H}_{3}\right)=0{.0345O}_{3}^{2}+14{.5O}_{3}+2650+0{.03H}_{3}^{2}+4{.2H}_{3}+0{.031O}_{3}H_{3}$	CHP type a *
30	$C_{c4}\left(O_{4}{,H}_{4}\right)=0{.0435O}_{4}^{2}{+36O}_{4}+1250+0{.027H}_{4}^{2}+0{.6H}_{4}+0{.011O}_{4}H_{4}$	CHP type b *
31	$C_{c5}\left(O_{5}{,H}_{5}\right)=0{.1035O}_{5}^{2}+34{.5O}_{5}+2650+0{.025H}_{5}^{2}+2{.203H}_{5}+0{.051O}_{5}H_{5}$	CHP type c *
32	$C_{c6}\left(O_{6}{,H}_{6}\right)=0{.072O}_{6}^{2}{+20O}_{6}+1565+0{.02H}_{6}^{2}+2{.34H}_{6}+0{.04O}_{6}H_{6}$	CHP type d *
33	$C_{c7}\left(O_{7}{,H}_{7}\right)=0{.0345O}_{7}^{2}+14{.5O}_{7}+2650+0{.03H}_{7}^{2}+4{.2H}_{7}+0{.031O}_{7}H_{7}$	CHP type a *
34	$C_{c8}\left(O_{8}{,H}_{8}\right)=0{.0435O}_{8}^{2}{+36O}_{8}+1250+0{.027H}_{8}^{2}+0{.6H}_{8}+0{.011O}_{8}H_{8}$	CHP type b *
35	$C_{c9}\left(O_{9}{,H}_{9}\right)=0{.0345O}_{9}^{2}+14{.5O}_{9}+2650+0{.03H}_{9}^{2}+4{.2H}_{9}+0{.031O}_{9}H_{9}$	CHP type a *
36	$C_{c10}\left(O_{10}{,H}_{10}\right)=0{.0435O}_{10}^{2}{+36O}_{10}+1250+0{.027H}_{10}^{2}+0{.6H}_{10}+0{.011O}_{10}H_{10}$	CHP type b *
37	$C_{c11}\left(O_{11}{,H}_{11}\right)=0{.1035O}_{11}^{2}+34{.5O}_{11}+2650+0{.025H}_{11}^{2}+2{.203H}_{11}$ $+0{.051O}_{11}H_{11}$	CHP type c *
38	$C_{c12}\left(O_{12}{,H}_{12}\right)=0{.072O}_{12}^{2}{+20O}_{12}+1565+0{.02H}_{12}^{2}+2{.34H}_{12}+0{.04O}_{12}H_{12}$	CHP type d *
39	$C_{h1}\left(T_{1}\right)=0{.038T}_{1}^{2}+2{.0109T}_{1}+950$	$0\;\leq {\;T}_{1}\;\leq \;2695.2$
40	$C_{h2}\left(T_{2}\right)=0{.038T}_{2}^{2}+2{.0109T}_{2}+950$	$0\;\leq {\;T}_{2}\;\leq \;60$
41	$C_{h3}\left(T_{3}\right)=0{.038T}_{3}^{2}+2{.0109T}_{3}+950$	$0\;\leq {\;T}_{3}\;\leq \;60$
42	$C_{h4}\left(T_{4}\right)=0{.052T}_{4}^{2}+3{.0651T}_{4}+480$	$0\;\leq {\;T}_{4}\;\leq \;120$
43	$C_{h5}\left(T_{5}\right)=0{.052T}_{5}^{2}+3{.0651T}_{5}+480$	$0\;\leq {\;T}_{5}\;\leq 120$
44	$C_{h6}\left(T_{6}\right)=0{.038T}_{6}^{2}+2{.0109T}_{6}+950$	$0\;\leq {\;T}_{1}\;\leq \;2695.2$
45	$C_{h7}\left(T_{7}\right)=0{.038T}_{7}^{2}+2{.0109T}_{7}+950$	$0\;\leq {\;T}_{2}\;\leq \;60$
46	$C_{h8}\left(T_{8}\right)=0{.038T}_{8}^{2}+2{.0109T}_{8}+950$	$0\;\leq {\;T}_{3}\;\leq \;60$
47	$C_{h9}\left(T_{9}\right)=0{.052T}_{9}^{2}+3{.0651T}_{9}+480$	$0\;\leq {\;T}_{4}\;\leq \;120$
48	$C_{h10}\left(T_{10}\right)=0{.052T}_{10}^{2}+3{.0651T}_{10}+480$	$0\;\leq {\;T}_{5}\;\leq 120$
	* See Appendix A.1.	

### Appendix A.4. 84-Unit Test System Data [30]

**POUs**							
**Unit No.**	**a_i_**	**b_i_**	**c_i_**	**d_i_**	**e_i_**	**P_i_^min^**	**P_i_^max^**
1–2	0.0069	6.73	94.705	100	0.084	36	114
3	0.02028	7.07	309.54	100	0.084	60	120
4	0.00942	8.18	369.03	150	0.063	80	190
5	0.0114	5.35	148.89	120	0.077	47	97
6	0.01142	8.05	222.33	100	0.084	68	140
7	0.00357	8.03	287.71	200	0.042	110	300
8	0.00492	6.99	391.98	200	0.042	135	300
9	0.00573	6.6	455.76	200	0.042	135	300
10	0.00605	12.9	722.82	200	0.042	130	300
11	0.00515	12.9	635.2	200	0.042	94	375
12	0.00569	12.8	654.69	200	0.042	94	375
13	0.00421	12.5	913.4	300	0.035	125	500
14	0.00752	8.84	1760.4	300	0.035	125	500
15–16	0.00708	9.15	1728.3	300	0.035	125	500
17	0.00313	7.97	647.85	300	0.035	220	500
18	0.00313	7.95	649.69	300	0.035	220	500
19	0.00313	7.97	647.83	300	0.035	242	550
20	0.00313	7.97	647.81	300	0.035	242	550
21–22	0.00298	6.63	785.96	300	0.035	254	550
23–24	0.00284	6.66	794.53	300	0.035	254	550
25–26	0.00277	7.1	801.32	300	0.035	254	550
27–29	0.52124	3.33	1055.1	120	0.077	10	150
30	0.0114	5.35	148.89	120	0.077	47	97
31–33	0.0016	6.43	222.92	150	0.063	60	190
34	0.0001	8.95	107.87	200	0.042	90	200
35–36	0.0001	8.62	116.58	200	0.042	90	200
37–39	0.0161	5.88	307.45	80	0.098	25	110
40	0.00313	7.97	647.83	300	0.035	242	550
CHP units							
Unit no.	α_i_	β_i_	γ_i_	δ_i_	ε_i_	ζ_i_	FOR
41–44, 49–52	0.0345	14.5	2650	0.03	4.2	0.031	CHP type a *
45–48, 53–56	0.0435	36	1250	0.027	0.6	0.011	CHP type b *
57–60	0.1035	34.5	2650	0.025	2.203	0.051	CHP type c *
61–64	0.072	20	1565	0.02	2.34	0.04	CHP type d *
HOUs							
Unit no.	η_i_	θ_i_	λ_i_	T_i_^min^		T_i_^max^	
65–68	0.038	2.0109	950	0		2695.2	
69–76	0.038	2.0109	950	0		60	
77–84	0.052	3.0651	480	0		120	
* See Appendix A.1.							

### Appendix A.5. 96-Unit Test System Data [30]

**POUs**							
**Unit No.**	**a_i_**	**b_i_**	**c_i_**	**d_i_**	**e_i_**	**P_i_^min^**	**P_i_^max^**
1, 14, 27, 40	0.00028	8.1	550	300	0.035	0	680
2, 3, 15, 16, 28–29, 41–42	0.00056	8.1	309	200	0.042	0	360
4–9,17, 18–22, 30–35, 43–48	0.00324	7.74	240	150	0.063	60	180
10–11, 23–24, 36–37, 49–50	0.00284	8.6	126	100	0.084	40	120
12–13, 25–26, 38–39, 51–52	0.00284	8.6	126	100	0.084	55	120
CHP units							
Unit no.	α_i_	β_i_	γ_i_	δ_i_	ε_i_	ζ_i_	FOR
53, 55, 59, 61, 65, 67, 71, 73	0.0345	14.5	2650	0.03	4.2	0.031	CHP type a *
54, 56, 60, 62, 66, 68, 72, 74	0.0435	36	1250	0.027	0.6	0.011	CHP type b *
57, 63, 69, 75	0.1035	34.5	2650	0.025	2.203	0.051	CHP type c *
58, 64, 70, 76	0.072	20	1565	0.02	2.34	0.040	CHP type d *
HOUs							
Unit no.	η_i_	θ_i_	λ_i_	T_i_^min^		T_i_^max^	
77, 82, 87, 92	0.038	0.01092	950	0		2695.2	
78–79, 83–84, 88–89, 93–94	0.038	0.01092	950	0		60	
80–81, 85–86, 90–91, 95–96	0.052	0.06513	480	0		120	
* See Appendix A.1.							
